# Supplementary material for: HUBMet: an integrative database and analytical platform for human blood metabolites and metabolite-protein associations
Source: Genome Biol. 2025 Dec 27;27:7. doi: 10.1186/s13059-025-03922-x (PMC12838415; doi:10.1186/s13059-025-03922-x)
Supplement: Supplementary file 2 — Additional file 2: Supplementary methods. Fig. S1. Data sources and evidence levels of metabolite-protein associations. Fig. S2. Annotation of metabolites and proteins in the network. Fig. S3. Tissue relevance criteria and summary of analysis result. Fig. S4. Differential analysis of proteins and metabolites, and network analysis in the case study. [file 13059_2025_3922_MOESM2_ESM.docx]

**Supplementary Materials for**

**HUBMet: an integrative database and analytical platform for human blood metabolites and metabolite-protein associations**

Xingyue Wang^1,2#^, Xiangyu Qiao^1,2#^, Alberto Zenere^1,2^, Swapnali Barde^1,2,3^, Jing Wang^1,2^, Wen Zhong^1,2,3*^

# Supplementary method

## Data collection and validation of blood metabolite-protein associations

Blood metabolite-protein associations were derived through an integration of three complementary data sources: (1) curated database, (2) human metabolic modelling, and (3) statistical correlations from two large-scale metabolomics and proteomics studies. First, we extracted 48,666 manually curated metabolite-protein associations between 1,491 metabolites and 1,905 proteins from HMDB (version 5.0). These associations were derived through systematic text mining and cross-validated against multiple references [1]. Second, we obtained 11,905 metabolic modelling-based associations between 516 metabolites and 2,643 proteins from the human genome-scale metabolic model (Human-GEM, accessed on December 12, 2023) [2]. Reaction equations used in metabolic modelling were obtained from GitHub repository (https://github.com/SysBioChalmers/Human-GEM/tree/main/model), and the metabolites and proteins involved in the same equation were identified as associated pairs. Third, we integrated statistical correlations from two large-scale, population studies that quantified matched blood metabolomes and proteomes [3,4]. The first study by Benson et al. employed Pearson correlation analysis and meta-analysis on proteome and metabolome data from 3,626 plasma samples across three human cohorts, including the Jackson Heart Study (JHS) [5], the Multi-Ethnic Study of Atherosclerosis (MESA) [6], and the Health, Risk Factors, Exercise Training and Genetics (HERITAGE) Family study [7], with comprehensive adjustments for age, sex, body mass index (BMI), and estimated glomerular filtration rate (eGFR) to account for potential confounding factors [3]. The second dataset from the Swedish SciLifeLab SCAPIS Wellness Profiling (S3WP) program applied linear mixed effect models with adjustments for age, sex, and BMI to characterize longitudinal plasma metabolome and proteome associations [4,8]. For both correlation studies, a threshold of < 0.001 for the Meta Q-value (Benson et al.) and the adjusted *p*-value (Wang et al.) was used, and 72,829 significant metabolite-protein correlations involving 545 metabolites and 1,186 proteins were identified. After integration and redundancy removal, a total of 129,814 unique associations between 4,455 proteins and 1,744 metabolites were identified.

We first validated these metabolite-protein associations using blood metabolome and proteome data from 182 control plasma samples in the Mayo Clinic Biobank (USA) [9,10]. Among 24,088 testable metabolite-protein associations in HUBMet (involving 588 metabolites and 700 proteins), 8,086 (33.6%) were reproducible at a *p*-value < 0.05. Further validation was conducted using the STITCH (Search Tool for Interacting Chemicals) database [11], which integrates chemical-protein interactions from publicly available databases (e.g., PDSP K_i_, PDB), literature, and computational predictions [12]. Although STITCH includes interactions beyond those specific to human blood, we applied stringent filtering criteria, selecting only experimentally supported interactions with medium or higher confidence scores (>400). Among 12,665 testable HUBMet associations involving 349 metabolites and 1,366 proteins, 2,804 (22.1%) were validated in STITCH.

## Data collection for the COVID-19 case study

Proteomics and metabolomics data used in the COVID-19 case study were obtained from a previously published retrospective cohort study conducted between April 14, 2020, and December 1, 2020 [9]. The study included 183 outpatients, 272 patients with severe or critical COVID-19, and 182 control individuals who visited one of three Mayo Clinic sites in the United States (Minnesota, Arizona, or Florida) during the study period.

## Protein annotation and gene expression

Protein annotations, including protein family, tissue specificity, functional class, and Gene Ontology terms, were downloaded from the Human Protein Atlas (HPA, version 23.0) and HMDB (version 5.0) [13,14]. The consensus normalized RNA expression levels for genes across tissues were downloaded from HPA.

## Metabolite set enrichment analysis

MSEA was performed in three main steps: (1) enrichment score calculation; (2) permutation test; and (3) multiple hypothesis testing correction.

Step 1: Enrichment score (*ES*) calculation

The full list of identified metabolites was ranked according to the Log2 fold changes (*FC*) between research groups (*L*). Correlation coefficients and other statistical measurements that can be used for ranking metabolite are also applicable. For each interested metabolite set (*S*), the running enrichment score (*RES*) was calculated starting with the metabolite (*m_i=1_*) with the highest *FC*. The *RES* was increased by the fraction of the *FC* of the hit metabolite (*|FC_j_|*) in the total absolute value of *FC* of the hit metabolites in *S* (*N_fc_*) when meeting a hit metabolite in the *S* and decreased when meeting a miss in the *S*, as showing in the following equation. *N* is the total number of metabolites in the list *L*; *N_H_* is the number of metabolites in the metabolite set *S*; *i* is the position in *L*.

$$RES_{hit}\left( S,i \right)=\sum_{m_{j}\in S, j\leq i} \frac{\left| FC_{j} \right|}{N_{fc}},\quad where N_{fc}=\sum_{m_{j}\in S} \left| FC_{j} \right|$$

$$RES_{miss}\left( S,i \right)=\sum_{m_{j}\notin S, j\leq i} \frac{1}{\left( N-N_{H} \right)}$$

The enrichment score for the metabolite set *ES(S)* was the maximum deviation from zero of *RES_hit_−RES_miss_*, which depended on both the *FC* and position of the metabolites in *S*.

Step 2: Estimating Significance

A permutation test was used to generate a null distribution of *ES* (*ES_null_*) for the interested metabolite set (*S*) and used for calculating nominal *p*-value. The same number of metabolites was randomly selected from the ranked list *L* for 1000 times and the *ES* of each random metabolite set for *S* was calculated. The nominal *p*-value for *S* is the positive or negative portion that are greater than or equal to *ES(S)* in positive or negative region of the null distribution.

Step 3: Multiple hypothesis testing

Enrichment score for both observed *ES(S)* and 1000 *ES* of permutation tests *ES(S,π)* was calculated by dividing by the mean of *ES(S,π)*. Positive and negative scores were scaled separately to get normalized enrichment scores *NES(S)* and *NES(S,π)*. *NES(S)* and *NES(S,π)* for each metabolite set (*S*) were recorded to generate two null distributions used for FDR calculation.

For a given metabolite set *S* with an observed *NES(S)* = *X* ≥ *0*, FDR is calculated by the ratio of the percentage of all the permutations with *NES(S,π)* ≥ *0*, whose *NES(S,π)* ≥ *X* divided by the percentage of all the *S* with the observed *NES(S)* ≥ *0*, whose  *NES(S)* ≥ *X*, and similar for a given metabolite set *S* with a observed *NES(S)* = *X* ＜ *0*.

$$\frac{N_{NES\left( S,\pi\right)\geq X}/N_{NES\left( S,\pi\right)\geq0}}{N_{NES\left( S \right)\geq X}/N_{NES\left( S \right)\geq0}}，when NES\left( S \right)=X\geq0$$

## References

1. Wishart DS, Tzur D, Knox C, Eisner R, Guo AC, Young N, et al. HMDB: the Human Metabolome Database. Nucleic Acids Res. 2007;35:D521-526.

2. Robinson JL, Kocabaş P, Wang H, Cholley P-E, Cook D, Nilsson A, et al. An atlas of human metabolism. Science Signaling. 2020;13:eaaz1482.

3. Benson MD, Eisman AS, Tahir UA, Katz DH, Deng S, Ngo D, et al. Protein-metabolite association studies identify novel proteomic determinants of metabolite levels in human plasma. Cell Metab. 2023;35:1646-1660.e3.

4. Tebani A, Gummesson A, Zhong W, Koistinen IS, Lakshmikanth T, Olsson LM, et al. Integration of molecular profiles in a longitudinal wellness profiling cohort. Nat Commun. 2020;11:4487.

5. Tahir UA, Katz DH, Zhao T, Ngo D, Cruz DE, Robbins JM, et al. Metabolomic Profiles and Heart Failure Risk in Black Adults: Insights From the Jackson Heart Study. Circ Heart Fail. 2021;14:e007275.

6. Bild DE, Bluemke DA, Burke GL, Detrano R, Diez Roux AV, Folsom AR, et al. Multi-Ethnic Study of Atherosclerosis: Objectives and Design. Am J Epidemiol. 2002;156:871–81.

7. Bouchard C, Leon AS, Rao DC, Skinner JS, Wilmore JH, Gagnon J. The HERITAGE family study. Aims, design, and measurement protocol. Med Sci Sports Exerc. 1995;27:721–9.

8. Wang J, Zenere A, Wang X, Bergström G, Edfors F, Uhlén M, et al. Longitudinal analysis of genetic and environmental interplay in human metabolic profiles and the implication for metabolic health. Genome Medicine. 2025;17:68.

9. Byeon SK, Madugundu AK, Garapati K, Ramarajan MG, Saraswat M, Kumar-M P, et al. Development of a multiomics model for identification of predictive biomarkers for COVID-19 severity: a retrospective cohort study. Lancet Digit Health. 2022;4:e632–45.

10. Olson JE, Ryu E, Hathcock MA, Gupta R, Bublitz JT, Takahashi PY, et al. Characteristics and utilisation of the Mayo Clinic Biobank, a clinic-based prospective collection in the USA: cohort profile. BMJ Open. 2019;9:e032707.

11. Szklarczyk D, Santos A, von Mering C, Jensen LJ, Bork P, Kuhn M. STITCH 5: augmenting protein–chemical interaction networks with tissue and affinity data. Nucleic Acids Res. 2016;44:D380–4.

12. Kuhn M, von Mering C, Campillos M, Jensen LJ, Bork P. STITCH: interaction networks of chemicals and proteins. Nucleic Acids Res. 2008;36:D684-688.

13. Wishart DS, Guo A, Oler E, Wang F, Anjum A, Peters H, et al. HMDB 5.0: the Human Metabolome Database for 2022. Nucleic Acids Res. 2022;50:D622–31.

14. Uhlén M, Fagerberg L, Hallström BM, Lindskog C, Oksvold P, Mardinoglu A, et al. Proteomics. Tissue-based map of the human proteome. Science. 2015;347:1260419.

# Supplementary figure


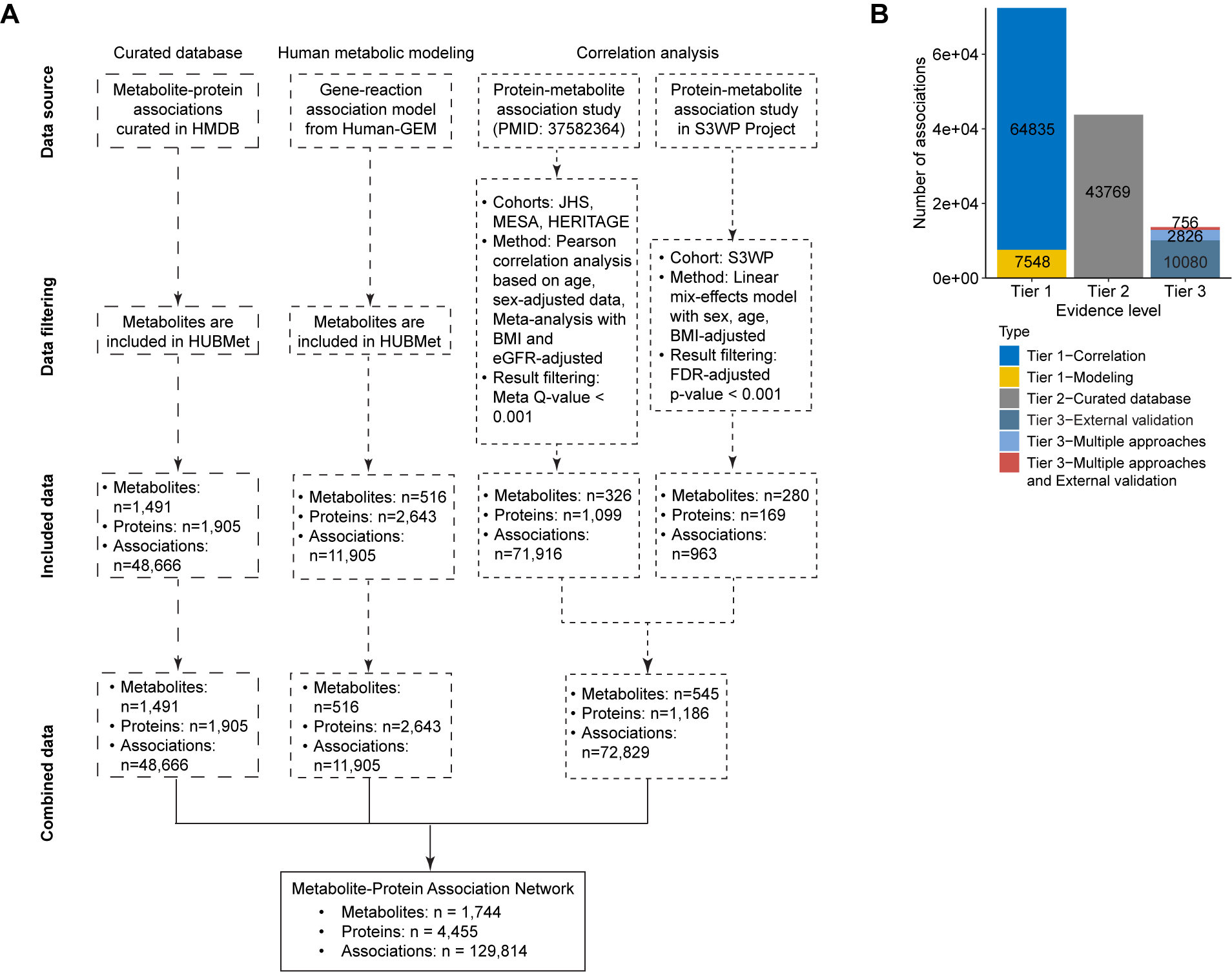


## Fig. S1 Data sources and evidence levels of metabolite-protein associations

**A** Detailed workflow for the collection of metabolite-protein association data. **B** Bar plot showing the number of metabolite-protein associations across three evidence levels. The color code indicates the criteria and supporting evidence.


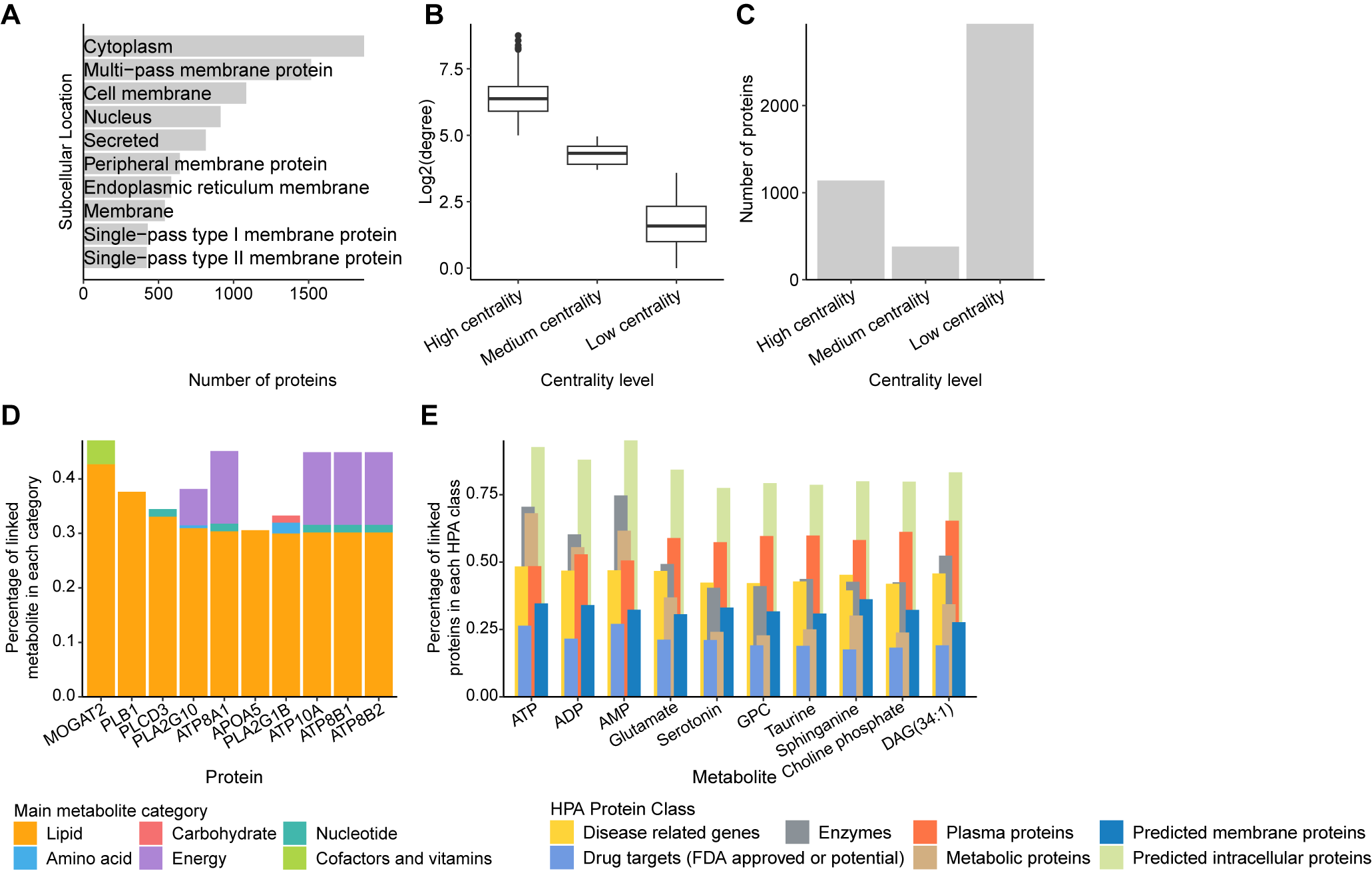


## Fig. S2 Annotation of metabolites and proteins in the network

**A** Bar plot showing the subcellular localization of the 1,744 metabolites included in the network. **B** Boxplot showing the distribution of protein degrees across three centrality levels. **C** Bar plot showing the number of proteins classified into three centrality level groups. **D** Bar plot showing the percentage of metabolite categories associated with the top 10 highest-centrality proteins in the network. **E** Bar plot showing the percentage of HPA protein classes among the top 10 hub metabolites with highest associated proteins.


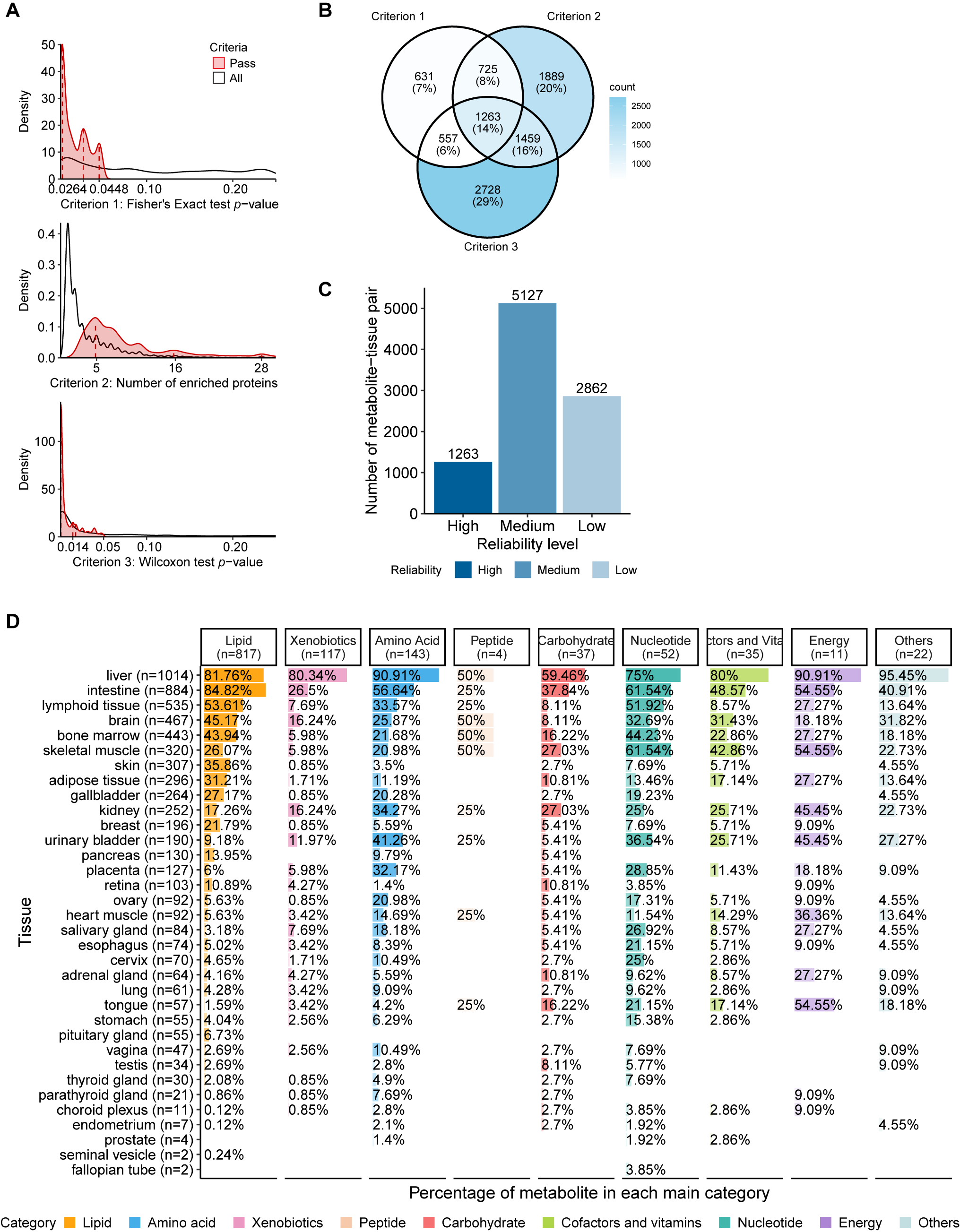


## Fig. S3 Tissue relevance criteria and summary of analysis result

**A** Density plots showing the distribution of test statistics for all evaluated metabolite-tissue pairs (black) and those meeting the defined criteria (red): *p*-values from Fisher’s exact test (top), number of enriched proteins (middle), and *p*-values from Wilcoxon test (bottom). **B** Venn plot illustrating the overlap of metabolite-tissue pairs that passed the different criteria, with colors indicating the number of overlapping pairs. **C** Bar plot showing the number of metabolite-tissue pairs classified across three reliability levels. **D** Bar plot showing the percentage of metabolites from each of the nine categories identified as relevant to 34 tissues. Only metabolite-tissue pairs with high or medium reliability are included.


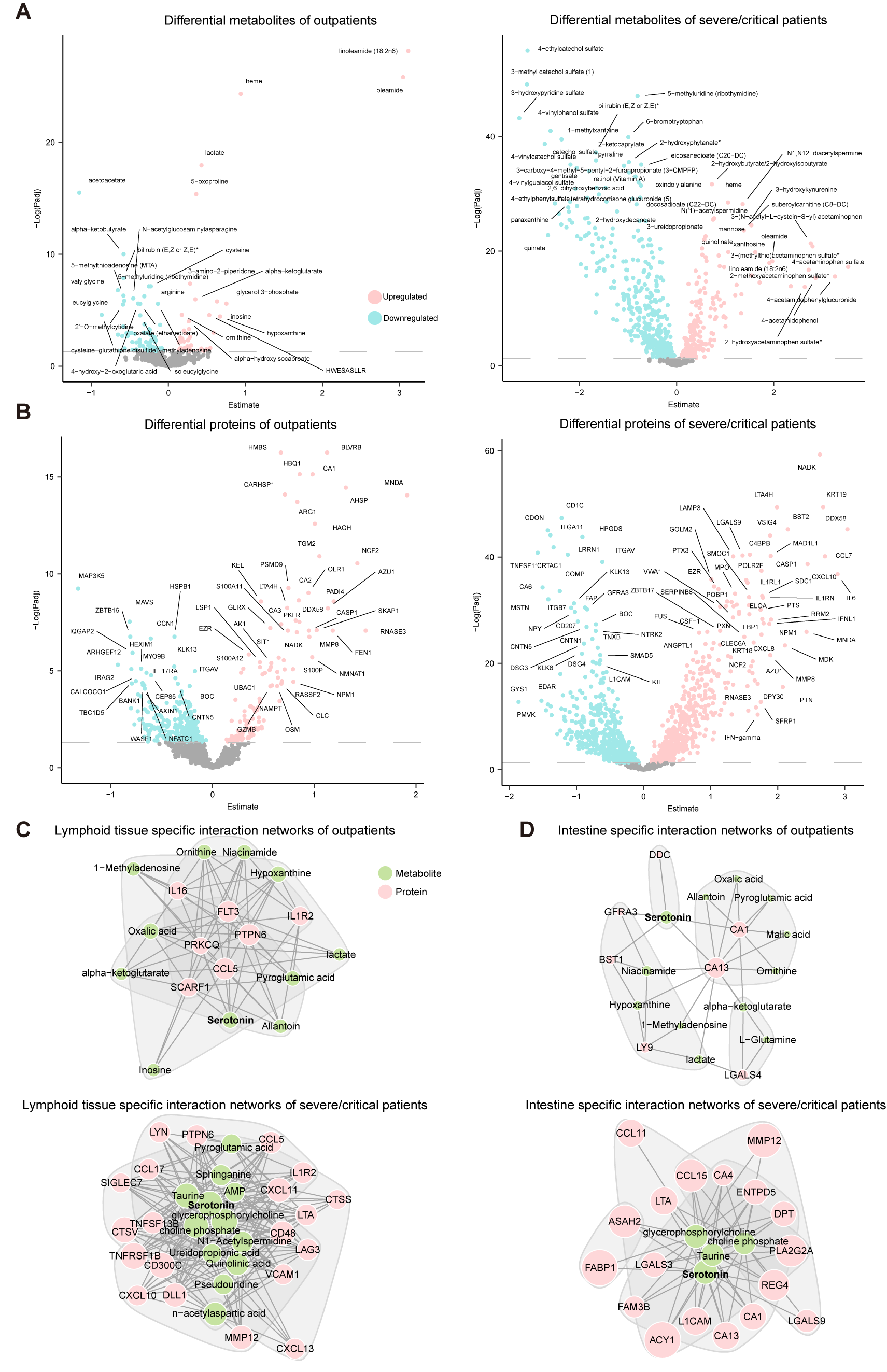


## Fig. S4 Differential analysis of proteins and metabolites, and network analysis in the case study

**A** Volcano plots showing differential metabolites between outpatient and control group (left), and severe/critical patient and control groups (right). The x-axis represents estimated coefficients, and the y-axis represents -Log10 *FDR*-adjusted *p*-value. The horizontal gray dashed line indicates the significance threshold at *FDR*-adjusted *p*-value less than 0.05. **B** Volcano plots showing differential proteins between outpatient and control group (left), and severe/critical patient and control group (right). **C** Association networks showing lymphoid tissue-associated differential metabolites and proteins in outpatients (top) and severe/critical patients (bottom) compared to controls. **D** Association networks showing intestine-associated differential metabolites and proteins for outpatients (top) and severe/critical patients (bottom) compared to controls.
